# Cerebral and cervical hemodynamic differences in adolescents with first-episode depressive disorder: a TCCD and cervical artery ultrasound study

**DOI:** 10.3389/fpsyt.2026.1892664

**Published:** 2026-08-13

**Authors:** Yihan Leng, Xinyue Wang, Xiaoqing Wang, Pingzi Wang, Jiaming Luo, Na Li, Litao Ye, Ling Li, You Yang, Yang Li, Jinhong Yu

**Affiliations:** 1 Department of Ultrasound Medicine, Affiliated Hospital of North Sichuan Medical College, Nanchong, Sichuan, China; 2 Mental Health Center, Affiliated Hospital of North Sichuan Medical College, Nanchong, Sichuan, China; 3 Department of Radiology, Affiliated Hospital of North Sichuan Medical College, Nanchong, Sichuan, China

**Keywords:** adolescents, cerebral hemodynamics, cervical artery ultrasound, first-episode depressive disorder, transcranial color-coded duplex sonography, vascular resistance

## Abstract

**Objective:**

To investigate cerebral and cervical hemodynamic differences assessed by transcranial color-coded duplex sonography (TCCD) combined with cervical artery ultrasound in adolescents with first-episode depressive disorder.

**Methods:**

This study included 169 adolescents with first-episode depressive disorder and 61 healthy controls. TCCD combined with cervical artery ultrasound was used to assess hemodynamic parameters of the main cerebral and cervical arteries, including peak systolic velocity, end-diastolic velocity, mean velocity, pulsatility index, and resistance index. Differences between the depressive disorder group and the healthy control group were compared. Associations between hemodynamic parameters and clinical characteristics, including Self-Rating Depression Scale (SDS) standard score, sex, age, and disease duration, were also analyzed.

**Results:**

The depressive disorder and healthy control groups were comparable in age [16 (13, 17) vs 16 (13.25, 17) years, *P* = 0.580] and sex distribution (female, 68.6% vs 62.3%, *P* = 0.367). Compared with healthy controls, adolescents with first-episode depressive disorder showed alterations in cerebral and cervical arterial hemodynamic parameters despite the absence of definite structural cerebrovascular lesions. The main changes were an increase in resistance-related indices in left middle cerebral artery, left anterior cerebral artery, and bilateral vertebral arteries. The velocity-related indices showed heterogeneous changes, including increased peak systolic velocity in the left anterior cerebral artery, decreased flow velocity in the intracranial segment of the right vertebral artery, and increased blood flow velocity in the cervical segment of the right internal carotid artery (all *P* < 0.05). Increasing age was predominantly associated with lower velocity-related parameters (all *P* < 0.05). Within the depressive disorder group, higher SDS standard scores were negatively associated with selected pulsatility, resistance, and velocity-related parameters after FDR correction (all FDR-adjusted *q* < 0.05). No consistent associations were found between hemodynamic parameters and sex or disease duration.

**Conclusion:**

TCCD combined with cervical artery ultrasound revealed differences in selected cerebral and cervical hemodynamic parameters in adolescents with first-episode depressive disorder, characterized by higher resistance-related indices and heterogeneous velocity-related changes. These findings suggest that combined ultrasound may provide complementary hemodynamic information and support future development of noninvasive hemodynamic characterization for adolescent depressive disorder.

## 1 Introduction

Depressive disorders represent a group of serious mental disorders with a high global prevalence and an increasing trend toward younger age of onset. Data from the World Health Organization indicate that about 3.8% of the global population is affected by depression (1). In China, this public health challenge is also relatively serious. A national epidemiological study reported that the lifetime prevalence of depressive disorders in adults was 6.8% (2), and the affected population continues to expand, underscoring the need for early identification and effective intervention in clinical medicine and public health (3). Clinically, depressive disorders are characterized primarily by marked and persistent depressed mood. Patients are often accompanied by anhedonia, cognitive impairment, substantial loss of energy, and pathological feelings of guilt (4). However, because their pathophysiological mechanisms have not yet been fully elucidated (5) and their clinical symptoms are highly heterogeneous, accurate diagnosis and individualized treatment remain challenging.

In recent years, mental health problems in children and adolescents have become more concerning, and studies have reported a growing burden of depression or depressive symptoms in this population (6, 7). WHO data reported that depressive disorders occur in 1.3% of individuals aged 10–14 years and in 3.4% of those aged 15–19 years (8). Because adolescence is an important stage for psychological and brain development, early recognition and timely intervention are needed for this age group (9).

At present, depressive symptoms and symptom severity are mainly evaluated using clinical interviews and standardized rating scales, such as the Hamilton Depression Rating Scale (HAMD) and the Self-Rating Depression Scale (SDS) (10, 11). However, these scales rely heavily on patient cooperation and self-awareness, while their results are susceptible to subjective concealment, exaggeration, or cognitive bias, which limits their objectivity to some extent. Moreover, the early symptoms of depressive disorders are often insidious, which may delay the timely identification of some potential patients and result in missed opportunities for optimal intervention. Therefore, exploring objective and quantifiable biological indicators (12) may help complement current symptom-based assessment methods and support individualized clinical evaluation.

Existing studies have demonstrated that depressive disorders are accompanied by various intracranial pathophysiological alterations (13), including impaired cerebral perfusion, disrupted electrophysiological activity, neuroendocrine dysfunction, and abnormalities in brain morphology and structure. The latter include hippocampal atrophy, thinning of the prefrontal cortex, abnormal functional connectivity within neural networks, and impaired integrity of white matter fiber tracts (14, 15). In clinical studies, several techniques have been used to examine brain abnormalities related to depression, including electroencephalography (EEG), magnetic resonance imaging (MRI), single-photon emission computed tomography (SPECT), and transcranial ultrasound (16–19). MRI and SPECT can provide useful information, but their application in routine assessment is limited by relatively high costs and long examination procedures. MRI also requires patients to stay in a confined and noisy environment, which may be difficult for some adolescents. These factors may cause psychological discomfort in patients, thereby limiting the clinical applicability of these techniques.

Transcranial ultrasonography offers a new direction for detecting intracranial abnormalities in patients with depressive disorders because of its advantages of being noninvasive, capable of real-time monitoring, easy to operate, and cost-effective. Previous evidence suggests that depressive disorders may be related to changes in cerebral hemodynamics (20). To better characterize cerebral hemodynamic changes, transcranial color-coded duplex sonography (TCCD), a noninvasive ultrasound technique for evaluating intracranial cerebral arteries, may provide useful vascular information. Compared with conventional transcranial Doppler sonography (TCD), TCCD combines two-dimensional grayscale imaging with color Doppler technology, allowing visual observation of intracranial vascular structures and blood flow status (21). In addition, TCCD provides imaging guidance for vascular localization and angle correction, which may improve the reliability of hemodynamic measurements. At present, TCCD studies on cerebral hemodynamic characteristics in patients with depressive disorders remain relatively limited. Most existing studies are constrained by insufficient sample sizes and substantial age heterogeneity among study participants. As a result, a systematic evidence base has not yet been established.

In this study, TCCD was used to assess blood-flow parameters of the main intracranial arteries in adolescents diagnosed with depressive disorders. The examined vessels included the middle cerebral artery (MCA; M1 segment), anterior cerebral artery (ACA; A1 segment), posterior cerebral artery (PCA; P1 segment), basilar artery (BA), and intracranial vertebral artery (VA; V4 segment). For these arteries, peak systolic velocity (PSV), end-diastolic velocity (EDV), mean velocity (MV), pulsatility index (PI), and resistance index (RI) were recorded, and these variables were further evaluated as exploratory hemodynamic indicators. Cervical arterial ultrasonography was performed at the same time to obtain corresponding measurements from the cervical portions of the vertebral artery (VA) and internal carotid artery (ICA). This study aimed to compare cerebral and cervical hemodynamic parameters between adolescents with first-episode depressive disorder and healthy controls, and to further examine age-related correlations and sex-based differences in both groups, as well as associations with SDS standard score and disease duration within the depressive disorder group.

## 2 Methods

### 2.1 Study design and participants

This was a single-center cross-sectional controlled observational study. It was conducted at the Mental Health Center of the Affiliated Hospital of North Sichuan Medical College from November 2024 to November 2025. A total of 169 adolescents with first-episode depressive disorder were recruited, including 53 males and 116 females, aged 10–19 years. In addition, 61 healthy controls were enrolled, including 23 males and 38 females, aged 10–19 years. Compared with adults, adolescents generally have thinner temporal bones and better acoustic permeability of the temporal window, which may help improve the stability of cerebral hemodynamic measurements.

All participants were diagnosed by experienced psychiatrists according to the ICD-10 criteria for depressive disorders. First-episode depressive disorder was defined as a first clinical diagnosis of depressive disorder without previous antidepressant treatment or related psychiatric intervention. After the diagnostic assessment, the Self-Rating Depression Scale (SDS) was used to evaluate depressive symptoms. The SDS contains 20 self-rated items, and each item is rated from 1 to 4. The total raw score ranges from 20 to 80. The standard score is obtained by multiplying the raw score by 1.25, with a higher score indicating more severe depressive symptoms. In this study, SDS scores were used to classify symptom severity and to compare different groups. According to the standard score, patients with depressive disorders were divided into mild depression (53–62 points), moderate depression (63–72 points), and severe depression (≥73 points).

### 2.2 Inclusion and exclusion criteria

Patients in the depressive disorder group were eligible if they met all of the following criteria: (1) a first clinical diagnosis of depressive disorder made by specialized psychiatrists according to the ICD-10 diagnostic criteria; (2) an SDS standard score ≥53; (3) no antidepressant treatment or other related intervention had been received before enrollment. Healthy controls were selected to be comparable with the depressive disorder group in age and sex, and their SDS standard scores were <53.

Participants in both groups were excluded if they met any of the following criteria: (1) organic lesions, including cerebrovascular disease or cervical arterial atherosclerotic plaques; (2) comorbid bipolar disorder or other psychiatric disorders; (3) a history of substance abuse or major unhealthy lifestyle factors, including smoking, alcohol abuse, obesity, or severe nutritional deficiency; (4) a definite family history of cardiovascular or cerebrovascular disease; (5) pregnancy or lactation.

### 2.3 Ethics approval and informed consent

The study protocol was reviewed and approved by the Institutional Medical Ethics Committee, with the approval number 2024ER546-1. All participants provided written informed consent before enrollment. For participants younger than 18 years, written informed consent was obtained from their legal guardians. All study procedures followed the Declaration of Helsinki and were performed under the guidance and supervision of psychiatrists.

### 2.4 Ultrasonographic assessment

Ultrasonographic examinations were performed using a Philips EPIQ 7 ultrasound system with an S5–1 phased-array probe (1.0–5.0 MHz) and an L12–3 linear-array probe (3.0–12.0 MHz). All participants were examined after resting for 10–15 minutes in a quiet environment to reduce stress-related interference and ensure the stability of baseline hemodynamic status. All measurements were performed repeatedly by one senior ultrasonographer with more than 10 years of experience in cardiovascular ultrasonography, strictly following the standardized measurement procedures described below. Each parameter was measured three times during the same examination session, and the mean value was used for statistical analysis to reduce random measurement variation. No fixed reference ranges were applied to classify individual hemodynamic measurements as normal or abnormal. The study was designed to assess group-level differences in hemodynamic parameters between adolescents with first-episode depressive disorder and healthy controls.

#### 2.4.1 TCCD examination of cerebral arteries

Participants were examined through the temporal acoustic window in the supine position. Imaging depth was adjusted according to head size until the contralateral skull echo was clearly visualized. The cerebral peduncle served as the key anatomical landmark (22). Bilateral MCA (M1), ACA (A1), and PCA (P1) were identified using color Doppler flow imaging (CDFI). For posterior circulation assessment, participants were repositioned in the prone position. The BA and intracranial VA (V4 segment) were visualized through the foramen magnum window.

For Doppler measurements, arterial segments with complete intraluminal color filling and clear vascular courses were selected. In pulsed-wave Doppler (PW) mode, the sample volume was placed within the lumen, and the gate size was adjusted according to vessel diameter. Pulse repetition frequency and color gain were optimized. The insonation angle was maintained below 60°, preferably ≤30° (23). After a stable spectral waveform was obtained, PSV, EDV, MV, PI, and RI were recorded.

#### 2.4.2 Cervical arterial ultrasonography

Participants were examined in the supine position using a linear-array probe to scan both sides of the neck. B-mode imaging was first used to confirm a smooth intima–media complex and the absence of atherosclerotic plaques in the cervical arteries. CDFI and PW modes were then applied to assess the cervical segments of the ICA and VA. During measurement, imaging depth, pulse repetition frequency, and color gain were optimized according to vessel depth and spectral waveform quality. The insonation angle was maintained below 60°. After a stable spectral waveform was obtained, PSV, EDV, MV, PI, and RI were recorded.

### 2.5 Statistical analysis

Data entry and statistical analysis were performed using IBM SPSS Statistics 27.0.1. The Shapiro–Wilk test was applied to examine whether continuous variables followed a normal distribution. Continuous variables with normal distribution were described as mean ± standard deviation (SD), while variables without normal distribution were expressed as median and interquartile range (IQR). Categorical variables were shown as frequencies and percentages.

Between-group comparisons were performed using the chi-square test for categorical variables and the independent-samples *t* test or Mann–Whitney *U* test for continuous variables, as appropriate. Difference estimates and 95% confidence intervals (95% CIs) were reported when applicable. Pearson or Spearman correlation analyses were used according to data distribution. Age-related correlations and sex-based comparisons were examined separately in the depressive disorder and healthy control groups. Within the depressive disorder group, associations of hemodynamic parameters with SDS standard score and disease duration were analyzed. For correlations between SDS standard score and 45 hemodynamic parameters, *P* values were adjusted using the Benjamini–Hochberg false discovery rate procedure, with FDR-adjusted *q* < 0.05 considered statistically significant.

Comparisons among healthy controls and depression severity subgroups were performed using one-way analysis of variance with Bonferroni *post hoc* tests, Welch’s analysis of variance with Games–Howell *post hoc* tests, or the Kruskal–Wallis *H* test with adjusted pairwise comparisons, as appropriate. Receiver operating characteristic analyses were conducted for hemodynamic parameters showing between-group differences. Severity-stratified and receiver operating characteristic analyses were considered exploratory. All tests were two-tailed, and *P* < 0.05 was considered statistically significant unless otherwise specified.

## 3 Results

### 3.1 Baseline and clinical characteristics

The study included 169 adolescents with first-episode depressive disorder, including 46 mild, 48 moderate, and 75 severe cases, and 61 healthy controls. Age and sex did not differ significantly between the two groups (both *P* > 0.05), suggesting that the two groups were comparable in baseline demographic characteristics (**Table 1**).

**Table 1:** Baseline and clinical characteristics of participants.

Variable	First-episode depressive disorder group	Healthy control group	Test statistic	*P* value
Sample size	169 (mild, 46; moderate, 48; severe, 75)	61	/	/
Sex, female, *n* (%)	116 (68.6%)	38 (62.3%)	χ² = 0.815	0.367
Age, years	16 (13, 17)	16 (13.25, 17)	*Z* = -0.553	0.580
SDS standard score	74.0 (64.5, 81.0)	33.0 (27.0, 42.0)	*Z* = -11.036	< 0.001
Disease duration, months	12 (4, 24)	/	/	/

Categorical variables are presented as *n* (%), and continuous variables are expressed as median (*P*25, *P*75). “/” indicates not applicable. A value of *P* < 0.05 was considered statistically significant.

### 3.2 Correlations between hemodynamic parameters and clinical characteristics

Correlations were used to examine the associations of age and SDS standard score with individual hemodynamic parameters. In the depressive disorder group, increasing age was associated with lower PSV in the left and right MCA, left ACA, left VA, and BA; lower EDV in the left ACA and BA; and lower MV in the left ACA, left VA, and BA (all *P* < 0.05). In healthy controls, increasing age was associated with lower PSV in the left and right MCA and left VA, lower EDV in the left MCA, and lower MV in the left and right MCA and left VA. By contrast, PSV and MV in the left ACA and RI in the right PCA increased with age (all *P* < 0.05).

In the depressive disorder group, higher SDS standard scores were associated with lower PI and RI in the bilateral MCA, lower PI and RI in the right ACA, and lower PSV in the left PCA and right VA (Spearman *ρ* = −0.278 to −0.375; all FDR-adjusted *q* < 0.05). No significant correlations were observed between disease duration and any measured hemodynamic parameter. Sex-based differences were limited to a small number of parameters and did not show a consistent hemodynamic pattern. The corresponding mean or median values, correlation coefficients, and statistical results are provided in **Supplementary Table 1**.

### 3.3 Between-group comparison of hemodynamic parameters

The depressive disorder group showed higher resistance-related indices in several cerebral arteries than the healthy control group, whereas velocity-related parameters showed heterogeneous changes. Specifically, PI was significantly higher in the left MCA, left ACA, and left VA in the depressive disorder group (all *P* < 0.05). RI was also higher in the left ACA and bilateral VAs (all *P* < 0.05). By contrast, the intracranial segment of the right VA showed lower velocity-related parameters, including significantly reduced PSV, EDV, and MV (all *P* < 0.05).

For cervical arteries, PI in the right VA was significantly higher in the depressive disorder group. EDV and MV in the right ICA were also higher in this group (all *P* < 0.05) (**Table 2**).

**Table 2:** Hemodynamic parameters showing significant between-group differences.

Parameter	Healthy controls (*n* = 61)	First-episode depressive disorder group (*n* = 169)	Difference estimate	95% CI	Test statistic	*P* value
Velocity-related hemodynamic parameters						
Cerebral artery						
PSV (ACA L) ^b^	74.60 (68.20, 88.70)	82.70 (75.05, 89.50)	-5.10	-8.90 to -0.90	*Z* = -2.476	0.013
PSV (VA R) ^b^	53.70 (48.50, 60.50)	50.40 (45.70, 55.45)	2.80	0.00 to 5.30	*Z* = -1.974	0.048
EDV (VA R) ^b^	25.20 (23.00, 29.00)	24.20 (19.60, 27.30)	2.30	0.50 to 4.20	*Z* = -2.418	0.016
MV (VA R) ^b^	35.53 (31.80, 38.93)	33.23 (27.98, 36.38)	2.80	0.73 to 4.33	*Z* = -2.646	0.008
Cervical artery						
EDV (ICA R) ^a^	35.63 ± 6.59	39.49 ± 8.96	-3.86	-6.21 to -1.50	*t* = 3.244	0.002
MV (ICA R) ^b^	58.80 (54.95, 64.47)	65.07 (55.73, 75.27)	-4.93	-9.23 to -1.07	*Z* = -2.468	0.014
Resistance-related hemodynamic parameters						
Cerebral artery						
PI (MCA L) ^b^	0.920 (0.800, 1.030)	1.020 (0.867, 1.120)	−0.070	-0.120 to -0.010	*Z* = -2.270	0.023
PI (ACA L) ^b^	0.870 (0.790, 1.080)	1.050 (0.880, 1.205)	−0.090	-0.160 to -0.020	*Z* = -2.384	0.017
RI (ACA L) ^b^	0.550 (0.516, 0.630)	0.619 (0.557, 0.668)	−0.033	-0.061 to -0.006	*Z* = -2.433	0.015
PI (VA L) ^b^	0.750 (0.670, 0.850)	0.810 (0.685, 0.970)	−0.080	-0.140 to -0.020	*Z* = -2.398	0.016
RI (VA L) ^b^	0.499 (0.462, 0.540)	0.528 (0.469, 0.594)	−0.036	-0.062 to -0.008	*Z* = -2.350	0.019
RI (VA R) ^b^	0.521 (0.446, 0.578)	0.541 (0.480, 0.594)	−0.029	-0.055 to 0.000	*Z* = -1.995	0.046
Cervical artery						
PI (VA R) ^a^	1.193 ± 0.177	1.271 ± 0.283	−0.077	-0.147 to -0.007	*t* = 2.180	0.031

Difference estimates were calculated as healthy control group minus the depressive disorder group. Negative values indicate higher values in the depressive disorder group. Superscript^a^ indicates normally distributed variables, presented as mean ± standard deviation with mean differences and 95% CIs. Superscript^b^ indicates non-normally distributed variables, presented as median (*P*25, *P*75) with Hodges–Lehmann differences and 95% CIs. Absolute *t* values and standardized *Z* values are reported as appropriate. All *P* values were two-sided and not adjusted for multiple comparisons. PSV, peak systolic velocity; EDV, end-diastolic velocity; MV, mean velocity; PI, pulsatility index; RI, resistance index; ACA, anterior cerebral artery; MCA, middle cerebral artery; VA, vertebral artery; ICA, internal carotid artery; L, left; R, right.

### 3.4 ROC analysis of differential hemodynamic parameters

Receiver operating characteristic (ROC) analysis showed that 10 of the 13 evaluated hemodynamic indicators yielded AUC values ≥ 0.60, indicating weak but statistically significant discriminative ability for distinguishing adolescents with first-episode depressive disorder from healthy controls (all *P* < 0.05). Among these indicators, MV in the right VA showed the largest AUC, although its discriminative utility remained limited (AUC = 0.631; 95% CI, 0.545–0.716; *P* = 0.003). The other indicators showed similar weak discriminative performance, with AUCs ranging from 0.607 to 0.626. These indicators included EDV in the right ICA and right VA; PI in the left VA, left ACA, and left MCA; RI in the left VA and left ACA; PSV in the left ACA; and MV in the right ICA.

Three indicators had AUC values < 0.60. PSV and PI in the right VA were statistically significant but showed very limited discriminative utility. RI in the right VA was not statistically significant (AUC = 0.598; 95% CI, 0.496–0.700; *P* = 0.059) (**Table 3**).

**Table 3:** ROC analysis of differential hemodynamic parameters.

Parameter	AUC	Standard error	95% CI	*P* value
Velocity-related hemodynamic parameters				
MV (VA R)	0.631	0.044	0.545 to 0.716	0.003
EDV (ICA R)	0.626	0.042	0.544 to 0.708	0.003
EDV (VA R)	0.619	0.043	0.534 to 0.704	0.006
PSV (ACA L)	0.617	0.047	0.525 to 0.709	0.013
MV (ICA R)	0.615	0.044	0.529 to 0.701	0.008
PSV (VA R)	0.597	0.048	0.503 to 0.691	0.042
Resistance-related hemodynamic parameters				
PI (VA L)	0.618	0.048	0.525 to 0.711	0.013
RI (VA L)	0.616	0.047	0.523 to 0.708	0.014
RI (ACA L)	0.615	0.047	0.522 to 0.708	0.015
PI (ACA L)	0.613	0.048	0.520 to 0.706	0.018
PI (MCA L)	0.607	0.049	0.511 to 0.703	0.030
PI (VA R)	0.593	0.043	0.508 to 0.678	0.033
RI (VA R)	0.598	0.052	0.496 to 0.700	0.059

AUC values and 95% CIs were calculated based on ROC curve analysis. Within each category, statistically significant parameters were arranged according to their AUC values. RI (VA R) was retained in the table for completeness, although it was not statistically significant. PSV, EDV, and MV are reported in cm/s, whereas PI and RI are dimensionless. AUC, area under the receiver operating characteristic curve; CI, confidence interval; PSV, peak systolic velocity; EDV, end-diastolic velocity; MV, mean velocity; PI, pulsatility index; RI, resistance index; ACA, anterior cerebral artery; MCA, middle cerebral artery; VA, vertebral artery; ICA, internal carotid artery; L, left; R, right.

### 3.5 Severity-related hemodynamic differences

Exploratory comparisons among healthy controls and the mild, moderate, and severe depression subgroups showed differences in selected velocity-related and resistance-related hemodynamic parameters across the anterior and posterior circulation (**Supplementary Table 2**; **Supplementary Figure 1**). Given the limited sample sizes within individual severity subgroups and the modest magnitude of the observed differences, detailed subgroup results and pairwise *P* values are provided in the **Supplementary Material**.

## 4 Discussion

As a cross-sectional observational study, this study primarily reflects cerebral hemodynamic characteristics at a single time point in first-episode, drug-naive adolescents with depressive disorder. The findings suggest that TCCD combined with cervical artery ultrasound may provide complementary hemodynamic information in this population.

The results showed differences in selected cerebral and cervical hemodynamic parameters in these adolescents, despite the absence of definite structural cerebrovascular lesions. A notable finding was the higher PI and RI observed in selected cerebral arteries, particularly the left MCA, left ACA, and left VA. This pattern is partially consistent with the findings of Gong et al. (24), who observed similar cerebral hemodynamic changes using TCD. This high-resistance pattern may be related to activation of the hypothalamic–pituitary–adrenal (HPA) axis and increased sympathetic tone (25). Dysregulated neurotransmitter metabolism and stress-related neuroendocrine changes may affect vascular regulation and contribute to functional constriction of intracranial arterioles (26). Persistent sympathetic activation and catecholamine accumulation may also be associated with increased cerebral vascular tone. In addition, Chiappelli et al. (27) reported that psychological stress burden was associated with altered regional cerebral blood flow, which provides indirect support for the relationship between stress-related mechanisms and cerebral perfusion changes.

Alongside these resistance-related differences, velocity-related parameters showed heterogeneous changes across vascular territories. Specifically, velocity was increased in certain arteries, such as the left ACA, but decreased in others, such as the right VA. This regional heterogeneity cannot be fully explained by vasoconstriction alone, underscoring the pathophysiological complexity of cerebral hemodynamic alterations in depressive disorder. When vascular resistance increases, cerebral autoregulation (CA) may help maintain relatively stable cerebral blood flow (CBF) (28). However, previous studies have shown that endothelial dysfunction, reduced nitric oxide bioavailability, and increased inflammatory cytokines may impair the vasomotor function of small cerebral vessels in patients with depressive disorder, leading to a significant reduction in cerebrovascular reactivity (CVR) (29, 30). Reduced CVR reserve may decrease vascular compliance and further compromise CA, thereby impairing the maintenance of cerebral perfusion homeostasis (31). Dysregulation of this autoregulatory mechanism may partly explain the heterogeneous changes in blood flow velocity observed in this study. Under high-resistance conditions, brain regions involved in different neurocognitive functions may exhibit marked spatial heterogeneity in hemodynamic responses because of regional differences in arterial distribution density (32, 33). When impaired microcirculation fails to dynamically compensate for neuronal activity, neurovascular coupling may be disrupted, resulting in a mismatch between neuronal metabolic demand and blood supply (34). Impaired microcirculatory regulation may also affect neurovascular coupling and contribute to affective and cognitive dysfunction (35).

Sex-based differences were limited to a small number of parameters and did not show a consistent hemodynamic pattern. Disease duration was not significantly correlated with the measured hemodynamic parameters. Age was significantly associated with several hemodynamic parameters in both groups, with most significant associations showing inverse correlations with flow velocity parameters. This finding may reflect age-related developmental changes in cerebral blood velocity during adolescence and is generally consistent with age-related changes in reference values reported in previous large-scale TCD studies (36).

Within the depressive disorder group, higher SDS standard scores were associated with lower pulsatility and resistance indices in the bilateral middle cerebral arteries and right anterior cerebral artery, as well as lower peak systolic velocity in the left posterior cerebral artery and right vertebral artery. These consistent inverse associations suggest that greater depressive symptom burden may be accompanied by differences in cerebrovascular hemodynamic regulation, including changes in vascular tone, neurovascular coupling, or regional cerebral perfusion. Previous evidence suggests that depression may involve impaired neurovascular coupling, inflammatory activity, and stress-related vascular alterations (37–39). These mechanisms may provide a plausible context for the observed associations.

Although exploratory subgroup analyses suggested that some hemodynamic parameters differed across depression severity categories (**Supplementary Table 2**; **Supplementary Figure 1**), these findings should be interpreted cautiously because of the limited sample sizes within individual subgroups and the modest magnitude of the observed differences. The current results are insufficient to establish a severity-dependent hemodynamic pattern.

Given the anatomical continuity between extracranial and intracranial vessels, this study additionally evaluated the hemodynamic characteristics of the cervical segments of the ICA and VA. These cervical arterial segments also showed concurrent abnormalities, including increased resistance in the right VA and altered flow velocities in the right ICA. These findings suggest that extracranial vascular alterations may coexist with intracranial hemodynamic changes. However, owing to the inherent limitations of ultrasound, including acoustic window attenuation and two-dimensional imaging constraints, future studies using advanced neuroimaging techniques with higher spatial resolution, such as 4D-flow MRI (40), are warranted to further clarify the interaction mechanisms and pathophysiological evolution of intracranial and extracranial hemodynamic changes in patients with depressive disorder.

## 5 Limitations

This single-center cross-sectional study cannot establish causality or characterize longitudinal hemodynamic changes. Although examinations were standardized and performed by one experienced ultrasonographer, technical and physiological variation, including acoustic-window dependence, probe angulation, and stress during examination, cannot be fully excluded. The modest between-group differences and the limited sample sizes of the healthy control and depression severity subgroups warrant cautious interpretation. In addition, the exploratory ROC findings were derived from the present single-center cohort and showed limited standalone discriminatory performance. Their potential clinical relevance requires confirmation in independent cohorts.

## 6 Conclusion

TCCD combined with cervical artery ultrasound identified differences in selected cerebral and cervical hemodynamic parameters in adolescents with first-episode depressive disorder compared with healthy controls. These differences were characterized by higher resistance-related indices and heterogeneous velocity-related changes. Within the depressive disorder group, higher SDS standard scores were negatively associated with selected resistance-related and velocity-related hemodynamic parameters. The findings provide preliminary evidence that cerebral and cervical hemodynamics may be associated with first-episode depressive disorder and may inform future research on noninvasive hemodynamic characterization in adolescent depressive disorder.

## Data Availability

The raw data supporting the conclusions of this article are not publicly available to protect participant confidentiality and privacy. De-identified data is available from the corresponding author upon reasonable request. Requests to access the datasets should be directed to yujinhong@nsmc.edu.cn.

## References

[1] World Health Organization . Depressive Disorder (Depression). Available online at: https://www.who.int/news-room/fact-sheets/detail/depression (Accessed May 17, 2026).

[2] Huang Y Wang Y Wang H Liu Z Yu X Yan J . Prevalence of mental disorders in China: a cross-sectional epidemiological study. Lancet Psychiatry. (2019) 6:211–24. doi: 10.1016/S2215-0366(18)30511-X 30792114

[3] Mekonen T Chan GCK Connor JP Hides L Leung J . Estimating the global treatment rates for depression: a systematic review and meta-analysis. J Affect Disord. (2021) 295:1234–42. doi: 10.1016/j.jad.2021.09.038 34665135

[4] American Psychiatric Association . DSM-5-TR. In: Diagnostic and Statistical Manual of Mental Disorders. Washington, DC: American Psychiatric Association Publishing (2022). doi: 10.1176/appi.books.9780890425787

[5] Malhi GS Mann JJ . Depression. Lancet. (2018) 392:2299–312. doi: 10.1016/S0140-6736(18)31948-2 30396512

[6] Xiang AH Martinez MP Chow T Carter SA Negriff S Velasquez B . Depression and anxiety among US children and young adults. JAMA Netw Open. (2024) 7:e2436906. doi: 10.1001/jamanetworkopen.2024.36906 39352699 PMC11445688

[7] Lu B Lin L Su X . Global burden of depression or depressive symptoms in children and adolescents: a systematic review and meta-analysis. J Affect Disord. (2024) 354:553–62. doi: 10.1016/j.jad.2024.03.074 38490591

[8] World Health Organization . Mental Health of Adolescents. Available online at: https://www.who.int/news-room/fact-sheets/detail/adolescent-mental-health (Accessed May 17, 2026).

[9] Davey CG McGorry PD . Early intervention for depression in young people: a blind spot in mental health care. Lancet Psychiatry. (2019) 6:267–72. doi: 10.1016/S2215-0366(18)30292-X 30502077

[10] Hamilton M . A rating scale for depression. J Neurol Neurosurg Psychiatry. (1960) 23:56–62. doi: 10.1136/jnnp.23.1.56 14399272 PMC495331

[11] Zung WWK . A self-rating depression scale. Arch Gen Psychiatry. (1965) 12:63. doi: 10.1001/archpsyc.1965.01720310065008 14221692

[12] García-Gutiérrez MS Navarrete F Sala F Gasparyan A Austrich-Olivares A Manzanares J . Biomarkers in psychiatry: concept, definition, types and relevance to the clinical reality. Front Psychiatry. (2020) 11:432. doi: 10.3389/fpsyt.2020.00432 32499729 PMC7243207

[13] Dean J Keshavan M . The neurobiology of depression: an integrated view. Asian J Psychiatry. (2017) 27:101–11. doi: 10.1016/j.ajp.2017.01.025 28558878

[14] Schmaal L Veltman DJ Van Erp TGM Sämann PG Frodl T Jahanshad N . Subcortical brain alterations in major depressive disorder: findings from the ENIGMA Major Depressive Disorder working group. Mol Psychiatry. (2016) 21:806–12. doi: 10.1038/mp.2015.69 26122586 PMC4879183

[15] Kaiser RH Andrews-Hanna JR Wager TD Pizzagalli DA . Large-scale network dysfunction in major depressive disorder: a meta-analysis of resting-state functional connectivity. JAMA Psychiatry. (2015) 72:603. doi: 10.1001/jamapsychiatry.2015.0071 25785575 PMC4456260

[16] Han S Li X-X Wei S Zhao D Ding J Xu Y . Orbitofrontal cortex-hippocampus potentiation mediates relief for depression: a randomized double-blind trial and TMS-EEG study. Cell Rep Med. (2023) 4:101060. doi: 10.1016/j.xcrm.2023.101060 37263267 PMC10313932

[17] Binnewies J Nawijn L Van Tol M-J Van Der Wee NJA Veltman DJ Penninx BWJH . Associations between depression, lifestyle and brain structure: a longitudinal MRI study. NeuroImage. (2021) 231:117834. doi: 10.1016/j.neuroimage.2021.117834 33549761

[18] Richieri R Boyer L Farisse J Colavolpe C Mundler O Lancon C . Predictive value of brain perfusion SPECT for rTMS response in pharmacoresistant depression. Eur J Nucl Med Mol Imaging. (2011) 38:1715–22. doi: 10.1007/s00259-011-1850-9 21647787

[19] Bei H-Z Chen J-P Mao C-J Zhang Y-C Chen J Du Q-Q . Echogenicity changes in brainstem raphe detected by transcranial parenchymal sonography and clinical characteristics in Parkinson’s disease. Front Neurol. (2020) 11:821. doi: 10.3389/fneur.2020.00821 32849249 PMC7426486

[20] Chithiramohan T Parekh JN Kronenberg G Haunton VJ Minhas JS Panerai RB . Investigating the association between depression and cerebral haemodynamics—a systematic review and meta-analysis. J Affect Disord. (2022) 299:144–58. doi: 10.1016/j.jad.2021.11.037 34800572

[21] Nedelmann M Stolz E Gerriets T Baumgartner RW Malferrari G Seidel G . Consensus recommendations for transcranial color-coded duplex sonography for the assessment of intracranial arteries in clinical trials on acute stroke. Stroke. (2009) 40:3238–44. doi: 10.1161/STROKEAHA.109.555169 19661474

[22] AIUM Practice Parameter for the Performance of Transcranial Doppler Ultrasound . AIUM practice parameter for the performance of transcranial doppler ultrasound. J Ultrasound Med. (2023) 42:E36–E44. doi: 10.1002/jum.16234 37132485

[23] Du L Hua Y Jia L Yang J Jiao L Liu J . Analysis of blood flow velocity variability in middle cerebral artery stenosis using transcranial color-coded sonography. J Clin Neurosci. (2025) 133:111020. doi: 10.1016/j.jocn.2024.111020 39787903

[24] Gong K Li Y Rong J Song J Ren F . Transcranial doppler ultrasound in evaluating cerebral blood flow abnormalities in major depressive disorder. Medicine. (2024) 103:e39889. doi: 10.1097/MD.0000000000039889 39432650 PMC11495716

[25] Leistner C Menke A . Hypothalamic–pituitary–adrenal axis and stress. In: Handbook of Clinical Neurology. Amsterdam: Elsevier (2020). p. 55–64. doi: 10.1016/B978-0-444-64123-6.00004-7 33008543

[26] Troubat R Barone P Leman S Desmidt T Cressant A Atanasova B . Neuroinflammation and depression: a review. Eur J Neurosci. (2021) 53:151–71. doi: 10.1111/ejn.14720 32150310

[27] Chiappelli J Adhikari BM Kvarta MD Bruce HA Goldwaser EL Ma Y . Depression, stress and regional cerebral blood flow. J Cereb Blood Flow Metab. (2023) 43:791–800. doi: 10.1177/0271678X221148979 36606600 PMC10108192

[28] Claassen JAHR Thijssen DHJ Panerai RB Faraci FM . Regulation of cerebral blood flow in humans: physiology and clinical implications of autoregulation. Physiol Rev. (2021) 101:1487–559. doi: 10.1152/physrev.00022.2020 33769101 PMC8576366

[29] Osimo EF Pillinger T Rodriguez IM Khandaker GM Pariante CM Howes OD . Inflammatory markers in depression: a meta-analysis of mean differences and variability in 5,166 patients and 5,083 controls. Brain Behav Immun. (2020) 87:901–9. doi: 10.1016/j.bbi.2020.02.010 32113908 PMC7327519

[30] Liu M He E Fu X Gong S Han Y Deng F . Cerebral blood flow self-regulation in depression. J Affect Disord. (2022) 302:324–31. doi: 10.1016/j.jad.2022.01.057 35032508

[31] Darling AM Richey RE Akins JD Saunders EFH Matthew Brothers R Greaney JL . Cerebrovascular reactivity is blunted in young adults with major depressive disorder: the influence of current depressive symptomology. J Affect Disord. (2021) 295:513–21. doi: 10.1016/j.jad.2021.08.061 34509066 PMC8667006

[32] Shaw K Bell L Boyd K Grijseels DM Clarke D Bonnar O . Neurovascular coupling and oxygenation are decreased in hippocampus compared to neocortex because of microvascular differences. Nat Commun. (2021) 12:3190. doi: 10.1038/s41467-021-23508-y 34045465 PMC8160329

[33] Smith PJ Blumenthal JA Hinderliter AL Watkins LL Hoffman BM Sherwood A . Microvascular endothelial function and neurocognition among adults with major depressive disorder. Am J Geriatric Psychiatry. (2018) 26:1061–9. doi: 10.1016/j.jagp.2018.06.011 30093218 PMC6165686

[34] Iadecola C . The neurovascular unit coming of age: a journey through neurovascular coupling in health and disease. Neuron. (2017) 96:17–42. doi: 10.1016/j.neuron.2017.07.030 28957666 PMC5657612

[35] Taylor WD Aizenstein HJ Alexopoulos GS . The vascular depression hypothesis: mechanisms linking vascular disease with depression. Mol Psychiatry. (2013) 18:963–74. doi: 10.1038/mp.2013.20 23439482 PMC3674224

[36] Alwatban MR Aaron SE Kaufman CS Barnes JN Brassard P Ward JL . Effects of age and sex on middle cerebral artery blood velocity and flow pulsatility index across the adult lifespan. J Appl Physiol. (2021) 130:1675–83. doi: 10.1152/japplphysiol.00926.2020 33703940 PMC8285608

[37] Yang X Wang J Zhang J Zhang M Hao A Guo F . Cerebrovascular-mediated dynamic alterations in neurovascular coupling: a key pathological mechanism of depression. Cell Biosci. (2025) 15:97. doi: 10.1186/s13578-025-01444-4 40624522 PMC12232600

[38] Samuels JD Lotstein ML Lehmann ML Elkahloun AG Banerjee S Herkenham M . Chronic social defeat alters brain vascular-associated cell gene expression patterns leading to vascular dysfunction and immune system activation. J Neuroinflamm. (2023) 20:154. doi: 10.1186/s12974-023-02827-5 37380974 PMC10303797

[39] Setiawan E Wilson AA Mizrahi R Rusjan PM Miler L Rajkowska G . Role of translocator protein density, a marker of neuroinflammation, in the brain during major depressive episodes. JAMA Psychiatry. (2015) 72:268. doi: 10.1001/jamapsychiatry.2014.2427 25629589 PMC4836849

[40] Sträter A Huber A Rudolph J Berndt M Rasper M Rummeny E . 4D-flow MRI: technique and applications. Fortschr Röntgenstr. (2018) 190:1025–35. doi: 10.1055/a-0647-2021 30103237

