# Supplementary material for: Cerebral and cervical hemodynamic differences in adolescents with first-episode depressive disorder: a TCCD and cervical artery ultrasound study
Source: Front Psychiatry. 2026 Aug 13;17:1892664. doi: 10.3389/fpsyt.2026.1892664 (PMC13519545; doi:10.3389/fpsyt.2026.1892664)
Supplement: Supplementary file 1 [file Table1.docx]

Supplementary Material

# 1 Supplementary Table S1. **Associations of age, sex, SDS standard score, and disease duration with hemodynamic parameters**

Panel A. Correlations between age and hemodynamic parameters

| **Group** | **Parameter** | **Method** | **Correlation coefficient** | ***P* value** |
| --- | --- | --- | --- | --- |
| Depressive disorder | PSV (MCA L) | Spearman | ρ = -0.208 | 0.019 |
| Depressive disorder | PSV (MCA R) | Spearman | ρ = -0.206 | 0.021 |
| Depressive disorder | PSV (ACA L) | Spearman | ρ = -0.190 | 0.040 |
| Depressive disorder | EDV (ACA L) | Spearman | ρ = -0.193 | 0.037 |
| Depressive disorder | MV (ACA L) | Spearman | ρ = -0.233 | 0.011 |
| Depressive disorder | PSV (VA L) | Spearman | ρ = -0.220 | 0.022 |
| Depressive disorder | MV (VA L) | Spearman | ρ = -0.197 | 0.040 |
| Depressive disorder | PSV (BA) | Spearman | ρ = -0.372 | <0.001 |
| Depressive disorder | EDV (BA) | Spearman | ρ = -0.214 | 0.029 |
| Depressive disorder | MV (BA) | Spearman | ρ = -0.317 | 0.001 |
| Healthy controls | PSV (MCA L) | Spearman | ρ = -0.528 | <0.001 |
| Healthy controls | PSV (MCA R) | Spearman | ρ = -0.423 | 0.002 |
| Healthy controls | EDV (MCA L) | Spearman | ρ = -0.471 | <0.001 |
| Healthy controls | MV (MCA L) | Spearman | ρ = -0.560 | <0.001 |
| Healthy controls | MV (MCA R) | Spearman | ρ = -0.405 | 0.003 |
| Healthy controls | PSV (ACA L) | Pearson | *r* = 0.286 | 0.042 |
| Healthy controls | MV (ACA L) | Pearson | *r* = 0.301 | 0.032 |
| Healthy controls | RI (PCA R) | Spearman | ρ = 0.280 | 0.046 |
| Healthy controls | PSV (VA L) | Spearman | ρ = -0.345 | 0.019 |
| Healthy controls | MV (VA L) | Spearman | ρ = -0.325 | 0.027 |

Panel B. Sex-based differences in hemodynamic parameters

| **Group** | **Parameter** | **Male** | **Female** | **Test statistic** | ***P* value** |
| --- | --- | --- | --- | --- | --- |
| Depressive disorder | EDV (MCA L) | 50.90 ± 10.92 | 45.93 ± 10.68 | *t* = 2.653 | 0.009 |
| Depressive disorder | PSV (ACA R) | 87.00 (79.30, 97.10) | 81.00 (72.70, 87.80) | *Z* = 2.937 | 0.003 |
| Depressive disorder | PSV (PCA L) | 67.40 (62.80, 77.85) | 64.10 (56.45, 70.58) | *Z* = 2.374 | 0.018 |
| Depressive disorder | RI (BA) | 0.57 ± 0.08 | 0.55 ± 0.07 | *t* = 2.004 | 0.047 |
| Healthy controls | PSV (MCA R) | 120.05 ± 17.18 | 110.59 ± 9.95 | *t* = 2.278 | 0.030 |
| Healthy controls | PSV (ACA L) | 68.66 ± 9.48 | 81.86 ± 9.42 | *t* = -4.828 | <0.001 |
| Healthy controls | EDV (ACA L) | 29.46 ± 6.83 | 33.70 ± 6.20 | *t* = -2.273 | 0.027 |
| Healthy controls | MV (ACA L) | 42.53 ± 6.89 | 49.76 ± 5.93 | *t* = -3.961 | <0.001 |
| Healthy controls | EDV (VA R) | 23.74 ± 2.19 | 26.60 ± 4.54 | *t* = -2.846 | 0.007 |
| Healthy controls | PSV (BA) | 62.35 (58.53, 78.02) | 78.85 (72.17, 92.75) | *Z* = -3.308 | <0.001 |
| Healthy controls | EDV (BA) | 26.33 ± 4.41 | 36.38 ± 5.30 | *t* = -7.007 | <0.001 |
| Healthy controls | PI (BA) | 1.04 ± 0.22 | 0.87 ± 0.16 | *t* = 2.984 | 0.004 |
| Healthy controls | RI (BA) | 0.61 ± 0.08 | 0.55 ± 0.07 | *t* = 2.809 | 0.007 |

Panel C. **Correlations between SDS standard score and hemodynamic parameters in the depressive disorder group**

| **Parameter** | **value** | **Method** | **Correlation coefficient** | **FDR-adjusted *q* value** |
| --- | --- | --- | --- | --- |
| PI (MCA L) | 0.99 ± 0.16 | Spearman | ρ = -0.346 | 0.005 |
| PI (MCA R) | 0.93 (0.86, 1.04) | Spearman | ρ = -0.293 | 0.022 |
| RI (MCA L) | 0.59 ± 0.06 | Spearman | ρ = -0.375 | 0.003 |
| RI (MCA R) | 0.57 (0.54, 0.62) | Spearman | ρ = -0.300 | 0.022 |
| PI (ACA R) | 0.90 (0.80, 1.10) | Spearman | ρ = -0.300 | 0.023 |
| RI (ACA R) | 0.56 (0.52, 0.63) | Spearman | ρ = -0.310 | 0.022 |
| PSV (PCA L) | 64.30 (57.05, 72.80) | Spearman | ρ = -0.278 | 0.041 |
| PSV (VA R) | 50.40 (45.25, 55.00) | Spearman | ρ = -0.290 | 0.023 |

Panel A shows age-related correlations, Panel B shows sex-based comparisons, and Panel C shows correlations between SDS standard score and hemodynamic parameters in the depressive disorder group. Pearson and Spearman correlation coefficients are denoted as r and ρ, respectively. Positive and negative coefficients indicate increasing and decreasing parameter values with increasing age or SDS score, respectively. Normally distributed variables are presented as mean ± standard deviation and non-normally distributed variables as median (*P*25, *P*75). Sex-based comparisons were performed using the independent-samples *t* test or Mann–Whitney *U* test, as appropriate. All tests were two-sided. Panels A and B list parameters with unadjusted *P* < 0.05. For Panel C, *P* values were adjusted using the Benjamini–Hochberg false discovery rate procedure across all parameters; only associations with FDR-adjusted *q* < 0.05 are shown. Disease duration was also analyzed, but no significant associations were identified. PSV, peak systolic velocity; EDV, end-diastolic velocity; MV, mean velocity; PI, pulsatility index; RI, resistance index; ACA, anterior cerebral artery; MCA, middle cerebral artery; PCA, posterior cerebral artery; VA, vertebral artery; BA, basilar artery; L, left; R, right.

# 2 Supplementary Table S2. Comparison of hemodynamic parameters across depression severity groups

Panel A. Non-normally distributed hemodynamic parameters

| **Parameter** | **Healthy controls** | **Mild depression** | **Moderate depression** | **Severe depression** | ***H* value** | ***P* value** |
| --- | --- | --- | --- | --- | --- | --- |
| PSV (MCA L) | 112.00 (104.00, 124.50)^a^ | 133.00 (108.25, 182.50)^b^ | 112.00 (101.00, 130.00)^a^ | 116.00 (104.50, 127.00)^a^ | 15.387 | 0.002 |
| PSV (MCA R) | 113.00 (107.00, 124.00)^a^ | 127.50 (115.75, 144.00)^b^ | 113.00 (101.00, 125.00)^ab^ | 111.00 (104.00, 120.50)^a^ | 11.543 | 0.009 |
| PI (MCA L) | 0.890 (0.795, 0.995)^a^ | 1.140 (0.983, 1.220)^b^ | 1.000 (0.910, 1.050)^ab^ | 0.920 (0.820, 1.040)^a^ | 13.640 | 0.003 |
| PI (MCA R) | 0.930 (0.825, 1.015)^ab^ | 0.990 (0.918, 1.173)^b^ | 0.910 (0.860, 1.030)^ab^ | 0.930 (0.835, 1.035)^a^ | 10.067 | 0.018 |
| RI (MCA R) | 0.574 (0.532, 0.605)^ab^ | 0.596 (0.567, 0.658)^b^ | 0.568 (0.544, 0.622)^ab^ | 0.573 (0.539, 0.613)^a^ | 10.382 | 0.016 |
| MV (MCA L) | 72.73 (66.03, 75.93)^ab^ | 72.4 (67.77, 103.05)^b^ | 67.87 (61.00, 78.40)^a^ | 68.53 (64.22, 79.70)^ab^ | 8.441 | 0.038 |
| PI (ACA L) | 0.870 (0.780, 1.070)^a^ | 1.190 (1.023, 1.330)^b^ | 0.990 (0.820, 1.080)^a^ | 1.060 (0.815, 1.180)^ab^ | 14.030 | 0.003 |
| PI (ACA R) | 0.900 (0.820, 1.035)^a^ | 1.150 (0.990, 1.243)^b^ | 0.860 (0.810, 1.040)^a^ | 0.920 (0.835, 1.105)^a^ | 23.816 | < 0.001 |
| RI (ACA L) | 0.549 (0.513, 0.625)^a^ | 0.665 (0.609, 0.705)^b^ | 0.598 (0.539, 0.628)^a^ | 0.620 (0.527, 0.661)^ab^ | 13.554 | 0.004 |
| RI (ACA R) | 0.563 (0.529, 0.611)^a^ | 0.651 (0.598, 0.680)^b^ | 0.548 (0.527, 0.615)^a^ | 0.570 (0.535, 0.631)^a^ | 24.335 | < 0.001 |
| PSV (PCA L) | 64.30 (59.25, 66.95)^ab^ | 74.15 (63.48, 88.90)^b^ | 62.20 (53.70, 68.20)^a^ | 67.40 (58.30, 76.10)^ab^ | 8.968 | 0.030 |
| PSV (PCA R) | 66.50 (60.55, 79.70)^ab^ | 71.65 (64.20, 81.43)^b^ | 62.50 (56.30, 69.00)^a^ | 67.10 (60.55, 73.75)^ab^ | 9.238 | 0.026 |
| PSV (VA R) | 53.70 (47.70, 60.10)^a^ | 54.80 (49.18, 59.70)^a^ | 50.30 (46.60, 55.00)^ab^ | 50.40 (44.05, 54.80)^b^ | 11.610 | 0.009 |
| PI (VA L) | 0.750 (0.655, 0.840)^a^ | 0.920 (0.773, 0.988)^b^ | 0.700 (0.620, 0.820)^a^ | 0.760 (0.635, 0.970)^a^ | 12.627 | 0.006 |
| PI (VA R) | 0.750 (0.640, 0.940)^a^ | 0.940 (0.780, 1.093)^b^ | 0.710 (0.670, 0.900)^a^ | 0.810 (0.675, 0.940)^ab^ | 12.352 | 0.006 |
| RI (VA L) | 0.499 (0.456, 0.538)^a^ | 0.570 (0.509, 0.597)^b^ | 0.475 (0.437, 0.538)^ab^ | 0.507 (0.445, 0.587)^ab^ | 11.854 | 0.008 |
| RI (VA R) | 0.500 (0.446, 0.578)^a^ | 0.577 (0.513, 0.632)^b^ | 0.482 (0.471, 0.562)^a^ | 0.525 (0.467, 0.585)^ab^ | 12.338 | 0.006 |
| MV (VA R) | 35.53 (31.80, 38.93)^a^ | 34.43 (27.88, 38.72)^ab^ | 33.53 (31.40, 36.93)^ab^ | 33.53 (27.68, 36.38)^b^ | 10.975 | 0.012 |
| PI (BA) | 0.960 (0.790, 1.090)^ab^ | 1.015 (0.860, 1.113)^b^ | 0.880 (0.720, 1.010)^a^ | 0.860 (0.735, 0.960)^a^ | 12.867 | 0.005 |

Panel B. Normally distributed hemodynamic parameters

| **Parameter** | **Healthy controls** | **Mild depression** | **Moderate depression** | **Severe depression** | **Test statistic** | ***P* Value** |
| --- | --- | --- | --- | --- | --- | --- |
| RI (MCA L) | 0.580 ± 0.070^a^ | 0.620 ± 0.060^b^ | 0.610 ± 0.070^ab^ | 0.570 ± 0.050^a^ | 5.613 | 0.001 |
| EDV (ACA R) | 34.76 ± 7.14^a^ | 29.18 ± 6.25^b^ | 35.65 ± 8.97^a^ | 37.86 ± 8.73^a^ | 7.480 | < 0.001 |
| MV (ACA R) | 50.68 ± 7.89^ab^ | 46.62 ± 8.38^a^ | 51.09 ± 8.84^ab^ | 54.33 ± 11.41^b^ | 4.330 | 0.006 |
| EDV (VA R) | 25.48 ± 4.02^a^ | 23.50 ± 5.46^ab^ | 24.78 ± 4.60^ab^ | 22.46 ± 5.29^b^ | 3.860 | 0.011 |
| RI (BA) | 0.570 ± 0.080^ab^ | 0.600 ± 0.060^b^ | 0.550 ± 0.080^a^ | 0.540 ± 0.070^a^ | 4.473 | 0.005 |

Panel A lists non-normally distributed variables, presented as median (*P*25, *P*75), and overall comparisons were performed using the Kruskal–Wallis H test. Panel B lists normally distributed variables, presented as mean ± standard deviation, and overall comparisons were performed using one-way ANOVA or Welch ANOVA, as appropriate. Post hoc pairwise comparisons were adjusted using the Bonferroni method for non-normally distributed variables and using the Bonferroni or Games–Howell method for normally distributed variables, as appropriate. Different superscript letters within the same row indicate statistically significant differences between groups after correction (*P* < 0.05), whereas shared letters indicate no statistically significant difference. PSV, EDV, and MV are expressed in cm/s; PI and RI are dimensionless. PSV, peak systolic velocity; EDV, end-diastolic velocity; MV, mean velocity; PI, pulsatility index; RI, resistance index; ACA, anterior cerebral artery; MCA, middle cerebral artery; PCA, posterior cerebral artery; VA, vertebral artery; BA, basilar artery; L, left; R, right.
